# Multiple Sclerosis Relapse Following COVID-19 Vaccination: A Case Report and Literature Review

**DOI:** 10.7759/cureus.21374

**Published:** 2022-01-18

**Authors:** Saurabh Kataria, Sylvette Rogers, Usama Bilal, Haisum Baktashi, Romil Singh

**Affiliations:** 1 Neurology, Ochsner Louisiana State University Health Shreveport, Shreveport, USA; 2 Neurology and Neurocritical Care, University of Missouri Health Care, Columbia, USA; 3 Neurology, West Virginia University, Morgantown, USA; 4 Neurology, Caribbean Medical University, Atlanta, USA; 5 Internal Medicine, Shaikh Khalifa Bin Zayed Al-Nahyan Medical and Dental College, Lahore, PAK; 6 Neurology, Liaquat National Hospital and Medical College, Karachi, PAK; 7 Critical Care, Allegheny Health Network, Pittsburgh, USA

**Keywords:** mrna-based vaccine, sars-cov-2 pandemic, covid-19 vaccine, multiple sclerosis flare-up, multiple sclerosis

## Abstract

Mass vaccination against coronavirus disease 19 (COVID-19) has effectively controlled the pandemic and has been remarkably effective and safe. Reports of a few adverse events have been reported after post-marketing surveillance. We present a rare case of multiple sclerosis (MS) relapse in a female who presented with fatigue, involuntary eye movements, and numbness; autoimmunity following the COVID-19 vaccine has also been described. She was diagnosed with MS six years back and was in remission. She received her COVID-19 vaccine 18 days ago. Her clinical and radiological features confirmed the MS relapse. Her serology for COVID-19 immunoglobulin G (IgG) and IgM was positive, and she was managed with intravenous methylprednisolone and symptomatic management. Our case provides a possible association of vaccine-associated MS relapse; however, more evidence is warranted from future studies.

## Introduction

Vaccine-related disorders are becoming evident as the neurological impact of the severe acute respiratory syndrome coronavirus 2 (SARS-CoV-2) pandemic continues to grow [1]. The mass vaccination against coronavirus disease 19 (COVID-19) is an excellent accomplishment, and vaccines have been remarkably effective and safe [2]. However, safety concerns and adverse outcomes are also emerging that need immediate attention. Myalgia, paresthesia, headache, and dizziness are the most commonly reported neurological manifestations following vaccination against COVID-19 [3]. There are also reports of potential post-vaccination neurological disorders, including transverse myelitis, multiple sclerosis (MS), Bell’s palsy, and Guillain-Barre syndrome. COVID-19 vaccination has been launched globally, and severe neurological syndromes occurred concurrently with the vaccine, although causality could not be established with absolute certainty [4]. Even though MS and its relapse is a sporadic neurological disorder associated with SARS-CoV-2, a few cases of MS relapse after the COVID-19 vaccine have also been reported [5]. We report a case of MS relapse in a patient who had received the COVID-19 vaccine.

## Case presentation

A 57-year-old female presented with complaints of fatigue for the last two days. Fatigue was present throughout the day, and her condition worsened gradually, and she began suffering from tingling, numbness, and stiffness in her left upper and lower limbs. She also reported involuntary eye movements and blurry vision for the last six hours. She was diagnosed with MS six years back and reported only one relapse two years back. She was on interferon beta and was compliant with her medication, and no new lesions were noted on brain magnetic resonance imaging (MRI) on recent follow-up. She denied any history of fever, trauma, or a family history of neurological disease. She did not smoke, drink alcohol, and use illicit drugs. She received her second dose of Pfizer COVID-19 vaccine 18 days back and reported only injection site pain at that time.

**Figure 1:**
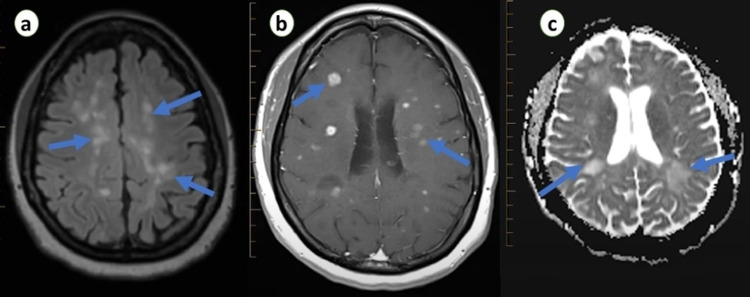
Transverse section of brain MRI showing white matter (a), juxtacortical (b), and paraventricular (c), deep lesions in various stages of enhancement in both hemispheres.

On examination, she was afebrile, anxious, and anicteric with a respiratory rate of 15 breaths/minute, heart rate of 80 beats/minutes, blood pressure of 130/90 mmHg, and oxygen saturation of 95%. Respiratory and cardiovascular examinations were unremarkable. On neurological examination, hyperreflexia and hypertonia were noted in the left upper and lower limbs. She had numbness and loss of vibrations in the lower limbs, predominantly on the left side, and her thought process was delayed. Eye examination revealed visual acuity of 100/200 in her left eye, and optic disc inflammation was observed on fundoscopic examination. The right eye was normal, and other cranial nerves were intact with no signs of meningeal irritation. Initial laboratory investigations were within normal limits. Brain MRI was performed, which revealed multiple confluent and distinct hyperintense white matter enhancing lesions in both hemispheres on T2-weighted and diffusion-weighted images (Figure 1). Spine MRI was normal, and cerebrospinal fluid analysis was non-significant except for elevated protein level. Her COVID-19 polymerase chain reaction (PCR) test was negative; however, the serology test was positive for SARS-CoV-2 immunoglobulin G (IgG) and IgM. Her infectious workup for human immunodeficiency virus, hepatitis B and C, and syphilis serology was negative as an infectious etiology of MS relapse. Her vitamin D level was also within the normal range.

She was managed with intravenous methylprednisolone, and physiotherapy was initiated. She was also given baclofen for stiffness and spasticity. She reported improvement in her vision, fatigue, and other neurological symptoms gradually. She was discharged on tapering oral steroids after four days with follow-up. Her lymphocyte count with subsets at the time of MS relapse and 24 days after vaccination are shown in Table 1.

**Table 1:** Lymphocytes and subsets during admission and 24 days after the vaccination. CD: cluster of differentiation

Lymphocytes and subsets	During admission	24 days after vaccination
White blood cell count	6100/mm^3^	5900/mm^3^
Lymphocytes	851 cell/uL	1003 cell/uL
CD3	580 cell/uL	619 cell/uL
CD4	419 cell/uL	531 cell/uL
CD8	98 cell/uL	141 cell/uL
CD19	83 cell/uL	115 cell/uL
CD20	92 cell/uL	115 cell/uL

## Discussion

While mass vaccination against coronavirus disease 19 (COVID-19) is an excellent accomplishment, safety concerns and adverse outcomes are also emerging. Adverse events after the COVID-19 vaccine include injection site reactions, myalgia, headache, fever, and asthenia [3]. Neurological syndromes, including new diagnosis of MS and transverse myelitis, have also been reported in the literature [6]. However, COVID-19 vaccine-induced MS relapse is not widely reported in the literature. Nistri et al. reported a case series in which 13 patients diagnosed with MS experienced MS relapse, among 2500 patients after mRNA COVID-19 vaccine and three patients diagnosed with MS. MS relapse was reported from three days to three weeks following SARS-CoV-2 vaccination. Disease reactivation was observed after both the first and second dose of the vaccine, and all the patients had signs of radiological activity on MRI to support the relapse [7]. Another case series reported nine cases of MS relapse after the AstraZeneca COVID-19 vaccine after a median interval of 13 days. All relapse cases were reported after the first dose of the COVID-19 vaccine and responded well to methylprednisolone [8]. Maniscalco et al. reported a case of MS relapse after the messenger ribonucleic acid (mRNA) COVID-19 vaccine. The patient reported weakness and paresthesia after 48 hours of the first dose of vaccine and responded well to methylprednisolone [9].

The potential association between MS relapse and the COVID-19 vaccine is still debated. A possible link between vaccine and MS activity has been suggested within 30 days following vaccination, given the possibility of vaccines enhancing the transition from subclinical to clinical disease after stimulating the immune response [10,11]. Immune response against vaccines may vary depending on the type of the vaccine and genetic susceptibility. Autoimmunity can be induced by the vaccine and its adjuvants, and autoimmune reaction occurs due to its cross-reactivity with structurally similar host proteins [12]. Cytokine upregulation, epitope spreading, and polyclonal activation of T and B lymphocytes induced by a vaccine can lead to autoimmunity by stimulating immune reactions [13]. In the case of adenoviral vaccines, the pathogenesis is related to adenovirus vectors, and the Pfizer COVID-19 vaccine is an mRNA vaccine encoding spike protein in lipid molecules with no adjuvants [14]. Hence, other mechanisms may also play a role in the pathogenesis of demyelinating diseases. A recent study noted that the interaction between host proteins and COVID-19 spike protein antibody and immunological interaction with myelin basic protein might contribute to the pathogenesis of demyelinating autoimmune diseases [12]. Additionally, angiotensin-converting enzyme 2 (ACE2) receptors are present in the endothelium of the blood-brain barrier or spinal neurons, and their interactions with viral spike protein can also trigger an inflammatory response [15].

Although many cases of MS relapse following the COVID-19 vaccine have been reported, causality cannot be determined. Furthermore, it is difficult to distinguish whether radiologically confirmed cases of relapses occurring after vaccination were triggered by the vaccine-induced inflammatory state or were MS relapses that would have manifested regardless of the COVID-19 vaccine [16]. At present, there are no contraindications for the COVID-19 vaccine in MS patients, and the only exception is the live-attenuated vaccine in patients who receive immunosuppressive or immunomodulatory therapy [17]. As infections can trigger a relapse, and infection risk outweighs the risk of vaccine-related adverse outcomes, vaccination in MS patients is not a contraindication; however, it is a reason to delay vaccination until remission. Therefore, the COVID-19 vaccine should be pursued as a general policy to decrease the risk of infection [18].

## Conclusions

Despite the excellent results of COVID-19 vaccination, safety concerns and monitoring of adverse outcomes must be addressed. Autoimmunity, especially MS relapse after administration of COVID-19 vaccine, has already been described in this study. Our case provides evidence of vaccine-associated MS relapse; however, the causality cannot be established in our patient based on radiologically confirmed MS relapse. In the wake of the mass vaccination against COVID-19, the data from future studies are warranted to establish the causality, and more cases of MS relapse are needed to confirm vaccine-related etiology.
